# A matrix‐assisted laser desorption ionization–time‐of‐flight–time‐of‐flight–mass spectrometry‐based toxicoproteomic screening method to assess in vitro particle potencies

**DOI:** 10.1002/jat.3642

**Published:** 2018-05-29

**Authors:** Marianne B. Ariganello, Dharani D. Das, Dalibor Breznan, Christine MacKinnon‐Roy, Fred Elisma, Aziz Khanchi, Renaud Vincent, Prem Kumarathasan

**Affiliations:** ^1^ Environmental Health Sciences and Research Bureau, HECSB, Health Canada Ottawa ON Canada K1A 0K9

**Keywords:** air particulate matter, air pollution, cytotoxicity, in vitro exposure, lung epithelial cells, macrophage, mass spectrometry, proteomics, toxicoproteomics

## Abstract

Knowledge of biological reactivity and underlying toxicity mechanisms of airborne particulate matter (PM) is central to the characterization of the risk associated with these pollutants. An integrated screening platform consisting of protein profiling of cellular responses and cytotoxic analysis was developed in this study for the estimation of PM potencies. Mouse macrophage (J774A.1) and human lung epithelial cells (A549) were exposed in vitro to Ottawa urban particles (EHC6802) and two reference mineral particles (TiO_2_ and SiO_2_). Samples from the in vitro exposure experiment were tested following an integrated classical cytotoxicity/toxicoproteomic assessment approach for cellular viability (CellTiter Blue®, lactate dehydrogenase) and proteomic analyses. Cellular proteins were pre‐fractionated by molecular weight cut‐off filtration, digested enzymatically and were analyzed by matrix‐assisted laser desorption ionization–time‐of‐flight–time‐of‐flight–mass spectrometry for protein profiling and identification. Optimization of detergent removal, pre‐fractionation strategies and enzymatic digestion procedures led to increased tryptic peptide (*m*/*z*) signals with reduced sample processing times, for small total protein contents. Proteomic analyses using this optimized procedure identified statistically significant (*P* < 0.05) PM dose‐dependent changes at the molecular level. Ranking of PM potencies based on toxicoproteomic analysis were in line with classical cytotoxicity potency‐based ranking. The high content toxicoproteomic approach exhibited the potential to add value to risk characterization of environmental PM exposures by complementing and validating existing cytotoxicity testing strategies.

## Abstract 2

To estimate in vitro potencies of ambient air particles we developed an integrated screening platform that enabled protein profiling of cellular responses and cytotoxic analyses. Extensive optimization of the toxicoproteomic analysis was carried out. Cellular proteomic changes were consistent with cytotoxicity findings. The integration of proteomic and cytotoxicity analyses results provided insight into particulate matter cytotoxic reactivity‐related mechanistic information at the molecular level.

## 1 INTRODUCTION

Ambient air particulate matter (PM) is a heterogeneous mixture of inorganic and organic components. There is increasing evidence associating ambient PM exposure with adverse health outcomes, including pulmonary, cardiovascular and neurological effects (Du, Xu, Chu, Guo, & Wang, 2016; Goldberg, Burnett, Yale, Valois, & Brook, 2006; Hajat, Haines, Goubet, Atkinson, & Anderson, 1999; Loane, Pilinis, Lekkas, & Politis, 2013; Pope 3rd et al., 2004). Air pollution is the fourth highest ranking risk factor for death, globally (Brauer et al., 2016). In addition, PM2.5 (particles with an aerodynamic diameter of less than 2.5 μm) is associated with larger global burden of disease (Brauer et al., 2016). There are also reports on molecular level changes related to adverse health consequences from air particle exposures (Breznan et al., 2016; Kumarathasan et al., 2015; Kumarathasan, Mohottalage, Goegan, & Vincent, 2005; Pope 3rd et al., 2004; Thomson et al., 2016; Vincent et al., 1997; Vincent et al., 2001; WHO Report, 2003).

At present, in vitro toxicity of PM is primarily evaluated using classical cellular level cytotoxicity endpoints. These assays allow for rapid, high‐throughput assessment of PM impacts on cell viability, metabolism and function in a cost‐effective manner. We have previously developed a high‐throughput method for similar cytotoxicity evaluation of PM and nanoparticles (Breznan et al., 2016; Breznan et al., 2017; Kumarathasan et al., 2015; Thomson et al., 2016), which allows to rapidly screen particles based on the viability of these cells. However, most significant changes due to particle exposures are observed with relatively higher doses of PM exposures, and these assays are limited in terms of providing detailed mechanistic information. Mechanistic information on PM toxicity at the molecular level can be achieved using high content “OMIC” analyses (e.g., protein or gene changes).

While toxicogenomic approaches have been recently recognized for their ability to contribute to understanding air pollution toxicity (Rager et al., 2011), toxicoproteomic approaches are still in their infancy, despite their ability to provide additional value to this area of research. The cellular proteome can by analyzed by various bioanalytical methods; one of which is two‐dimensional gel electrophoresis followed by mass spectroscopy (MS). Liquid chromatography coupled with MS (LC/MS) and matrix‐assisted laser desorption ionization MS (MALDI‐MS) are two additional approaches used for this purpose. While traditional two‐dimensional gel electrophoresis offers a technologically simple and lower cost approach in terms of analytical instrumentation, the process is time‐consuming and requires relatively larger amounts of protein in the sample to be tested (requiring large number of cells and increased quantities of particles for in vitro exposures). Furthermore, there can be large variations arising due to gel performance, reproducibility and the analyst (Baggerman, Vierstraete, De Loof, & Schoofs, 2005). Although both LC/MS and MALDI‐MS require more elaborate and costly analytical platforms and highly skilled personnel, these methods can be reproducible and automated (Roe & Griffin, 2006; Zhang, Wu, Stenoien, & Paša‐Tolić, 2014). Of these analytical platforms, advantages of using a MALDI‐based MS method (vs. LC‐based) include the use of almost no solvent, reduced analysis time and relatively easier interpretation of the MS data because of mostly singly charged ions formed during the MALDI process. In addition, the MALDI‐MS technique can handle a relatively large number of analyses without analyst intervention. While the higher resolution LC–MS (e.g., triple‐quad and Orbitrap) techniques can provide relatively large proteome coverage, the throughput may be limited due to the LC performance (retention time changes with larger number of analyses, relatively longer run time/sample) and its consequence on the MS analyses requires relatively frequent analyst intervention. In terms of particle toxicity screening studies, the number of samples generated will be very high and thus true high throughput and well blocked analyses have to be considered as key requirements.

In this work, the objective was to develop an optimized shot‐gun proteomic analysis method based on a MALDI‐TOF‐TOF‐MS platform to screen environmental particles for their toxicity characteristics. For this purpose, in vitro exposures of two separate cell lines (macrophages and epithelial cells) to an urban air particle and two reference mineral particles were carried out to analyze cellular and proteomic changes.

## 2 MATERIALS AND METHODS

Corning cell culture T‐75 culture flasks, 96‐well plates, Hyclone cell culture media (Dulbecco's modified Eagle's medium [DMEM]), fetal bovine serum (FBS), Halt Protease Inhibitor Cocktail (100×) and Pierce Detergent Removal Spin Columns (catalog no. P87777) were purchased from ThermoFisher Scientific (Waltham, MA, USA). Gentamicin, phosphate‐buffered saline, Triton X‐100 Tween‐80 and Trifluoroacetic acid (TFA) were obtained from Sigma‐Aldrich (Oakville, ON, Canada). Lactate dehydrogenase (LDH) cytotoxicity assay kits (CytoTox‐96®), CellTiter‐Blue®, ProteaseMax™ Surfactant Solution, Trypsin Gold, Trypsin/Lys C Mix (V5071) and chymotrypsin were from Promega Corporation (Madison, WI, USA). α‐Cyano‐4‐hydroxycinnamic acid was purchased from Bruker Daltonics (Billerica, MA, USA) and acetonitrile (ACN) from EMD (Etobicoke, ON, Canada). All water used was deionized/demineralized (>16 MΩ resistivity). Stock solutions of trypsin/Lys C and trypsin (50 μg ml^−1^ in freshly prepared 50 mm ammonium acetate [NH_4_OAc, pH 7.4]) and chymotrypsin (500 μg ml^−1^ in freshly prepared 50 mm acetic acid) were aliquoted and stored at −80°C, and were subjected to a maximum of two freeze/thaw cycles. Stock solutions of ProteaseMax (1% in freshly prepared 50 mm NH_4_HCO_3_) were aliquoted and stored at −25°C, and were subjected to a maximum of three freeze/thaw cycles. Matrix solutions were prepared fresh daily (5 mg ml^−1^ α‐Cyano‐4‐hydroxycinnamic acid in 50% ACN in 0.1% TFA in water).

### 2.1 Particle preparation

TiO_2_ (SRM‐154b) and SiO_2_ (SRM‐1879a) were obtained from the National Institute of Standards and Technology (Gaithersburg, MD, USA) and served as standard reference materials throughout the assessments. These materials were washed three times with methanol (15 seconds vortex, 1 minute sonication in ice bath, pelleted by centrifugation at 13 000 *g* for 10 minutes) then rinsed (centrifugation only) three times with particle buffer (0.19% NaCl and 25 μg ml^−1^ of the non‐ionic detergent Tween‐80) to remove possible soluble metals and organic contaminants before use in the experiments (Vincent et al., 1997). The EHC6802 urban air particles were recovered in the same manner as the EHC93 urban air particles (Vincent et al., 2001; Vincent, Goegan, et al., 1997). Particles were resuspended at 10 mg ml^−1^ in particle buffer, vortexed (30 seconds), sonicated (20 minutes in ice‐cold water bath) and homogenized with a Dounce homogenizer (25 strokes). Particle suspensions were aliquoted into sterile microcentrifuge tubes and heated at 56°C for 30 minutes, then stored at −40°C until use (Nadeau, Vincent, Kumarathasan, Brook, & Dufresne, 1996).

### 2.2 Physicochemical characterization of the particles

Scanning electron microscopy (SEM) was conducted to assess the morphology and size of EHC6802, SiO_2_ and TiO_2_ particles in dry state using the procedure reported (Das et al., 2014). The average particle size was determined from the mean size of at least 50 individual particles using NIH ImageJ 1.47 software (https://imagej.nih.gov/ij/download.html). The particles were assumed as spherical and the diameters of the sphere fitting the individual particles were recorded as their size. The elemental analysis of the above particles was carried out using inductively coupled plasma–MS method reported elsewhere (Breznan et al., 2017). Particle size and the agglomeration state in liquid (ultrapure water and DMEM +5% FBS media) were determined by dynamic light scattering (DLS), while the surface charge of the particle suspensions was assessed based on the electrokinetic (zeta) potential of the particles (Das et al., 2014). It should be noted that the DMEM +5% FBS is used as the cell exposure medium in all experiments. Particle concentrations of 0.05 and 0.16 mg ml^−1^ correspond to the doses of 30 and 100 μg cm^−2^ of well surface area, respectively.

### 2.3 In vitro exposure of cells to particles

Mouse macrophages (J774A.1) and human lung epithelial cells (A549), obtained from ATCC (Manassas, VA, USA), were subcultured in T75 flasks containing DMEM supplemented with 10% FBS and 50 μg ml^−1^ gentamicin, seeded in 96‐well plates at 40 000 cells per well or 20 000 cells per well, respectively and cultured for 24 hours before dosing with particles. Particle stocks were thawed at room temperature, vortexed (10 seconds), sonicated (10 minutes on ice) and diluted with serum‐free, phenol‐red free media and aliquots of these particle suspensions were used to dose cells in 96‐well plates at increasing doses (30, 100 and 300 μg cm^−2^) of TiO_2_ particles, EHC6802 and SiO_2_ particles for additional 24 hours (Kumarathasan et al., 2015). The final FBS concentration in each well was 5%. For the purposes of analyzing actual particulate samples, J774 and A549 cells exposed to 0, 30 and 100 μg cm^−2^ doses of the particles as outlined above were processed using the optimized method detailed below, which utilized detergent removal, sequential fractionation using molecular weight cut‐off (MWCO) filters and an overnight trypsin‐Lys C digestion.

### 2.4 Integrated classical cytotoxicity/toxicoproteomic analyses

#### 2.4.1 Cytotoxicity assays at the cellular level

Cell culture supernatants and cell lysates obtained after 24 hours of particle exposure were used to analyze cellular viability (cellular metabolism by resazurin reduction (CellTiter‐Blue®), cell membrane integrity (ratio of LDH release into the cell culture supernatant to total LDH activity), as reported previously (Kumarathasan et al., 2015). The remainder of the aliquoted cell lysates were stored at −80°C for proteomic analyses.

### 2.5 Toxicoproteomic analyses

#### 2.5.1 Sample preparation

Frozen cell lysate samples in 96‐well plates were thawed on ice, centrifuged for 15 minutes (900 *g*), and subsequently duplicate samples were pooled into sterile 600 μl tubes containing 10 μl of 10× protease inhibitor cocktail. Samples were refrozen at −80°C to subject cells to freeze–thaw conditions. On the day of sample processing for proteomic analysis, cell lysates were thawed on ice and sonicated for 20 minutes in an ice bath, then centrifuged at 10 000 *g* for 10 minutes. The supernatants were subsequently processed for detergent removal as described below.

#### 2.5.2 Optimization of detergent removal

Two methods (A and B) of detergent (Triton X‐100) removal were tested. In method A, clarified cell lysates were passed through either a 3 kDa or a 10 kDa pre‐wetted (with 300 μl dH_2_O) MWCO filter. The filtrate was discarded and the residue was diluted with the same volume of dH_2_O and passed through the same MWCO, repeating this process three to four times. In method B, clarified cell lysates were cleaned up using detergent removal spin columns (catalog no. P87777; Pierce) following the manufacturer's instructions. Briefly, columns were initially conditioned with 50 mm NH_4_OAc, clarified cell lysate samples were added subsequently and centrifuged at 1500 *g* for 2 minutes.

#### 2.5.3 Optimization of sample fractionation

After the removal of detergent, cell lysate samples were fractionated using different combinations of MWCO filtration processes to optimize sample fractionation. This was done to select a fractionation strategy that can generate a less complex matrix, as well as a faster pre‐fractionation process. The strategies were: (A) five‐filter sequence (3, 10, 30, 50 and 100 kDa); (B) three‐filter sequence (10, 50 and 100 kDa); and (C) four‐filter sequence (10, 30, 50 and 100 kDa). For all three protocols, detergent‐free cell lysate was diluted (1:3) with dH_2_O and loaded on to the smallest MWCO filter in that sequence. For instance, in strategy A, the sample was clarified using the 3 kDa filter set‐up by centrifuging at 14 000 *g* for 30 minutes at 4°C and the filtrate was collected. The residue from the filter was diluted with 200 μl dH_2_O, then inverted and spun at 5000 *g* for 3 minutes to collect the primary residue, the filter was rinsed again with 75 μl of 10% ACN/dH_2_O and spun into the same collection tube as the primary residue. The residue was then clarified on a 10 kDa MWCO filter set‐up and was spun at 14 000 *g* for 15 minutes at 4°C, the filtrate was saved and the residue was treated as mentioned above to clarify further on higher MWCO filters successively (30, 50 and 100 kDa) according to the specified fractionation strategy.

Three different residue collection methods were compared in this study to optimize protein recovery: (1) two‐step dissolution of residue with initial 200 μl dH_2_O followed by 75 μl of 10% ACN/dH_2_O; (2) dissolution of residue using 200 μl of 10% ACN/dH_2_O only; and (3) dissolution of residue using 50 μl of 50 mm NH_4_OAc (recommendation from the manufacturer). All filtrates and the final 100 kDa residue from the different MWCO fractionations were evaporated to dryness using a gentle stream of N_2_ and frozen at −80°C.

#### 2.5.4 Optimization of sample digestion

Dried samples were resuspended in 25 μl of 50 mm NH_4_OAc (pH 7) and were treated with the digestion enzyme. Three different digestion enzymes were compared: trypsin only (16.7 μg ml^−1^); trypsin combined with Lys C (16.7 μg ml^−1^); and chymotrypsin (23.5 μg ml^−1^). Samples were sonicated for 5 minutes in an ice bath, centrifuged for 1 minute at 5000 *g*, incubated overnight in a water bath at 37°C with parafilm wrap to prevent evaporation. Following the 18 hour enzyme digestion, reaction was quenched with 5 μl of 5% TFA in dH_2_O, vortexed for 3 seconds and centrifuged at 14 000 *g* for 10 minutes. Clarified samples were spotted on an AnchorChip MALDI target plate (600/384F; Bruker Daltonics Ltd, Bremen, Germany). In addition, once the optimal enzyme combination was selected based on the MS output, enzyme concentration was optimized (e.g., ±35%). The efficiency of enzyme digestion was further tested by assessing the effect of two different digestion buffers (50 mm Tris–HCl, pH 8.0 and 50 mm NH_4_OAc, pH 7.4) with or without 0.05% ProteaseMax™ based on MS data. Acid quenching and centrifugation also helped to remove any degradation products of the ProteaseMax™ detergent.

### 2.6 MALDI TOF‐TOF‐MS analysis

#### 2.6.1 Sample spotting

One μl of sample was spotted on an AnchorChip target plate (600/384F; Bruker Daltonics Ltd, Bremen, Germany) followed by the addition of 1 μl of freshly prepared 5 mg ml^−1^ α‐cyano‐4‐hydroxycinnamic acid. This mixture was gently mixed by pipetting up and down (Kumarathasan et al., 2005). Spots were dried at room temperature and were washed by applying 2.5 μl of ice‐cold 1% TFA in dH_2_O (10 seconds). Following spotting, the remaining digested samples were flash frozen on dry ice and stored at −80°C. Flash freezing the solubilized peptides was selected over drying the peptides to minimize potential problems of incomplete re‐solubilization due to the small volumes of liquid used for reconstitution. Multiple spotting was carried out per sample (*n* = 5 for MS scans and *n* = 3 for MS/MS analysis).

#### 2.6.2 Mass spectrometry

Each spot was analyzed by Bruker AutoFlex (III) MALDI‐TOF‐TOF‐MS platform (Bruker Daltonics Ltd, Bremen, Germany) in manual as well as automated mode. In addition, settings such as gain, pulsed ion extraction, laser power and number of laser shots were adjusted to improve peptide signals. The instrument was calibrated both at the beginning of a sample run and after every three spots using peptide‐II calibration standard (Bruker Daltonics, Billerica, MA, USA) for *m*/*z* accuracy. The MS analysis of each spot was performed to obtain a peptide *m*/*z* scan. Mass spectral data were queried using the bioinformatics software ClinPro Tools (Bruker Daltonics Ltd, Bremen, Germany) for candidate biomarkers (Kumarathasan et al., 2012). The candidate biomarker peptide peaks were subjected to subsequent MS/MS analysis in the “Lift” mode. The mass spectral information (MS/MS data only) was matched against SwissProt and NCBInR databases using the MASCOT search engine (Matrix Science, Boston, MA, USA) for protein identification.

### 2.7 Statistical analysis

Resazurin reduction and lactate dehydrogenase release values were normalized to the mean of the respective controls to generate fold‐change values for each particle dose. Classical cytotoxicity dose–response data were assessed for statistically significant effects by one‐way ANOVA compared to 0 Dose. Data sets not meeting the assumptions of normality and equal variance for ANOVA were rank‐transformed before analyses. Peptides included in the hierarchical clustering heat maps were selected based on the statistical tests performed by ClinPro Tools software (Bruker Daltonics Ltd, Bremen, Germany). In addition, peptide intensities were normalized within each biological replicate to their respective, “no treatment” controls. Subsequent comparisons of normalized peptide expressions for each particle type were performed within all pairs of doses using one‐sided paired *t*‐tests. For each pair of doses, one‐sided alternative hypotheses in both directions were tested.

Statistical analyses of cytotoxicity data were conducted using SigmaPlot version 13 (Systat Software, Inc., San Jose, CA, USA), while the normalized peptide expression data were analyzed for significance using R version 3.2.0 (Foundation for Statistical Computing, Vienna, Austria, https://cran.r-project.org/bin/windows/base/old/3.2.0/). Statistical significance was accepted at *P* < .05. Heatmap and hierarchical clustering software was used to visualize and cluster peptides (http://www.hiv.lanl.gov/content/sequence/HEATMAP/heatmap_mainpage.html; Los Alamos National Laboratory, Los Alamos, NM, USA).

### 2.8 Potency estimates

As a simplified description of the dose–effect relationship, potency estimates (β) of the particles were derived from the following equation: fold‐change = (Dose +1)^β^ where “β” is the rate of change of dose with respect to the log of fold‐effect for a given endpoint (Vincent, Goegan, et al., 1997). Dose–effect data were fitted using CurveExpert v1.3 (D. Hyams, Hixson, TN, USA) to obtain the “β” values.

## 3 RESULTS

### 3.1 Physicochemical properties of the particles

**Figure 1:**
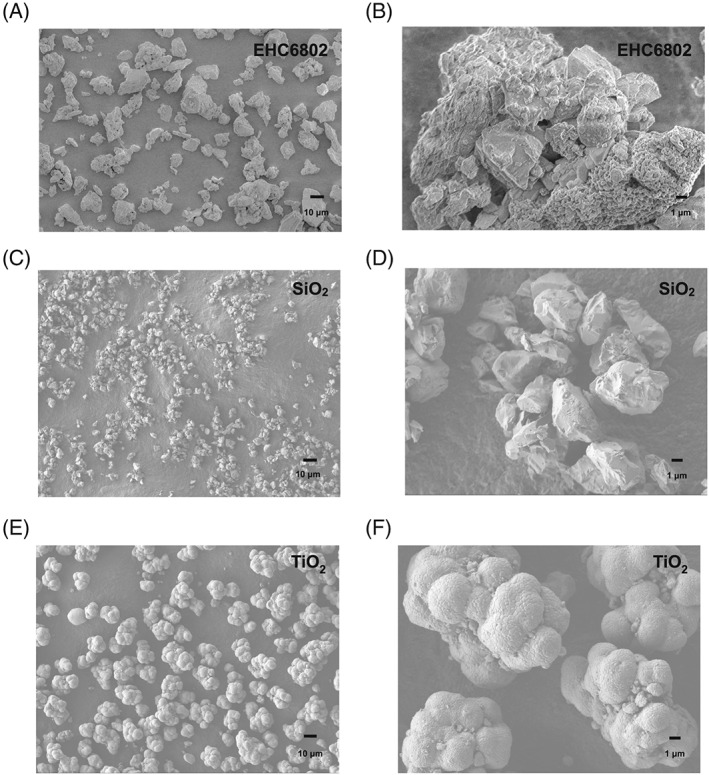
Scanning electron microscopy analysis results for the particles. (A,B) EHC‐6802 urban particulate material. (C,D) SiO_2_ standard reference material. (E,F) TiO_2_ standard reference material. Scale bars indicating images at two different magnifications (10 μm, 1 μm), shown in the lower left corner of each image

The electron micrographs in Figure 1 showed the morphology of the EHC6802 (Figure 1A,B), SiO_2_ (Figure 1C,D) and TiO_2_ (Figure 1E,F) particles in dry state. All three materials were complex mixtures of various size, shape and agglomeration/aggregation state. The EHC6802 showed the highest heterogeneity in shape and size, while SiO_2_ particles were of irregular shape and TiO_2_ formed large globular agglomerates with fused individual particles. Particle size distributions from SEM images revealed the mean diameter of 18.8 ± 5.0, 5.2 ± 1.8 and 18.9 ± 5.2 μm for EHC6802, SiO_2_ and TiO_2_, respectively.

The elemental analyses (Table 1) revealed that EHC6802 was dominated by aluminum, lead, magnesium, titanium and zinc, while SiO_2_ contained chromium, potassium and zirconium at levels >1000 ppm. Potassium and lead were found in TiO_2_ particles at relatively higher levels (>1000 ppm) in relation to the other measured elements.

**Table 1:** Elemental compositions of particles based on inductively coupled plasma–mass spectrometry analyses

Elements (ppm)	Particles		
	EHC6802	SiO_2_	TiO_2_
Ag	–	67.23	2.13
Al	19 417.33	2.05	1.23
As	–	4.86	3.25
B	–	557.25	137.03
Ba	446.29	17.25	52.38
Ca	–	0.62	1.84
Cd	–	2.49	3.74
Ce	–	33.06	1.11
Co	–	53.53	6.50
Cr	–	1075.50	26.74
Cs	–	0.25	0.22
Cu	811.96	0.36	0.04
Dy	–	6.46	0.16
Er	–	0.62	0.03
Eu	–	0.21	0.02
Fe	–	8.17	1.68
Ga	–	1.21	2.56
Gd	–	4.92	0.06
Hf	–	70.80	3.38
Ho	–	0.27	0.01
In	–	2.49	0.03
K	–	1348.82	1395.25
La	–	48.45	0.46
Li	–	6.30	2.57
Lu	–	0.07	0.005
Mg	15 503.73	0.21	0.35
Mn	462.64	119.17	8.19
Mo	13.05	150.96	18.12
Na	–	1.42	2.12
Nb	–	60.14	0.12
Nd	–	1.58	1.42
Ni	55.09	873.61	42.68
P	–	250.80	550.07
Pb	6010.26	36.79	1126.07
Pr	–	0.30	0.09
Rb	–	2.38	3.18
S	–	0.16	4.18
Sb	21.96	164.37	1.20
Sc	–	2.55	0.50
Sm	–	2.56	0.34
Sn	890.65	14.77	11.44
Sr	343.88	11.92	7.58
Tb	–	5.14	0.01
Th	–	6.94	3.84
Ti	1642.81	–	–
Tm	–	0.08	0.01
U	–	4.14	0.97
V	91.49	27.33	39.98
W	–	118.76	0.13
Y	–	9.96	0.29
Yb	–	0.97	0.03
Zn	5663.53	0.10	0.88
Zr	–	2422.27	81.47

The particles were also characterized in water and in DMEM +5% FBS using DLS (Table 2). All three materials were observed to agglomerate in both water and cell exposure media, as indicated by generally high polydispersity index (>0.5) of the particle suspensions. In addition, sedimentation was observed to occur for the particles throughout the measurements. The hydrodynamic diameter of all three particles increased with increased particle concentration in both water and cell exposure media. The three particles showed a decreasing trend in their hydrodynamic diameter in the serum‐containing medium compared to water at 0.05 mg ml^−1^ particle concentration. Similar trend was also observed for EHC6802 and SiO_2_ at 0.16 mg ml^−1^ particle concentration, with the exception of TiO_2_, which revealed a substantial increase in its hydrodynamic size in the cell exposure media, but it is associated with a relatively higher analytical variance (Table 2).

**Table 2:** Dynamic light scattering size and zeta potential of particles in water and DMEM +5% FBS

Particles	Particle concentration 0.05 mg ml^−1^		Particle concentration 0.16 mg ml^−1^	
	Particle hydrodynamic diameter (nm)	Zeta potential (mV)	Particle hydrodynamic diameter (nm)	Zeta potential (mV)
Water				
EHC6802	783 ± 280	−1.2 ± 0.7	1654 ± 392	−9.3 ± 0.5
SiO_2_	5103 ± 516	−53.0 ± 8.7	13730 ± 10144	−43.8 ± 5.1
TiO_2_	1113 ± 54	−0.3 ± 0.2	1070 ± 54	−11.6 ± 1.2
DMEM +5% FBS				
EHC6802	247 ± 165	−10.1 ± 1.5	469 ± 161	−10.6 ± 0.7
SiO_2_	309 ± 107	−0.2 ± 1.5	5912 ± 730	−9.8 ± 0.2
TiO_2_	373 ± 137	−9.2 ± 0.4	12730 ± 17141	−8.6 ± 0.9

DMEM, Dulbecco's modified Eagle's medium; FBS, fetal bovine serum.

Particle concentrations of 0.05 and 0.16 mg ml^−1^ correspond to the doses of 30 and 100 μg cm^−2^ of well surface area respectively.

The relative instability of the particle suspensions in both water and cell exposure media was further indicated by zeta potential values between 0 and − 15 mV (Table 2) in most preparations, with the exception of SiO_2_, which suggested a comparatively higher stability in water, with zeta potential value above −40 mV (0.05 and 0.16 mg ml^−1^).

### 3.2 Cytotoxicity

**Figure 2:**
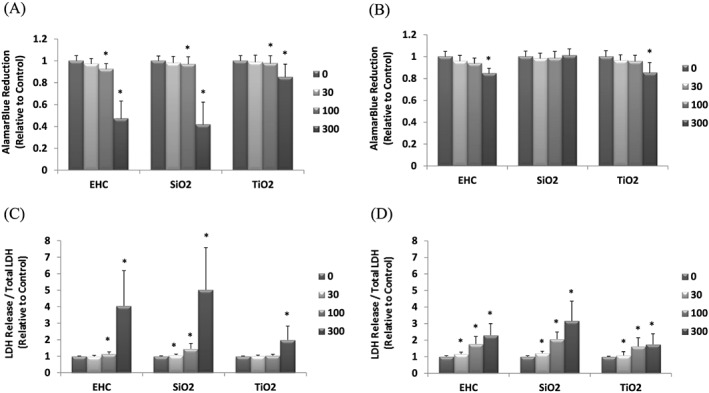
Cytotoxic responses in cells exposed to particulate matter for 24 h. Changes in resazurin reduction in J774 (A) and A549 (B) cells and LDH release in J774 (C) and A549 (D) cells, respectively. All columns denoted with * are significantly (*P* < 0.05) different from the control (0 dose). LDH, lactate dehydrogenase

Differential cytotoxicity responses were observed for all particles and both cell types. The results indicated higher average particle potency (β_Ave_) in general for SiO_2_ and EHC6802 for both J774 and A549 cells relative to TiO_2_. Nevertheless, relative potency estimates for TiO_2_ were comparable between the cell lines (Table 3). Overall, J774 cells were more sensitive in their response to all particle exposures compared to A549 cells (Figure 2). SiO_2_ showed the greatest toxicity overall for both cell types. J774 cells exposed to SiO_2_ showed the greatest decrease in resazurin reduction at both 100 and 300 μg cm^−2^ doses, while TiO_2_ exhibited the least change (Figure 2A, B). A similar pattern was found for LDH release (Figure 2C,D) and cellular LDH content (data not shown) for both cell types. For A549 cells, a minor decrease in resazurin reduction was observed following treatment with the highest doses of both EHC6802 and TiO_2_ particles.

**Table 3:** Particle rankings based on cytotoxic and proteomic potency estimates (β)

	Cytotoxicity				Proteomics	
	β_LDH Rel_	β_Resaz Reduct_	β_Ave_ a	Rank	β_Ave_ b	Rank
A. J774 Potencies						
SiO_2_	0.22	−0.056	0.14	1	0.037	1
EHC	0.17	−0.045	0.11	2	0.030	2
TiO_2_	0.073	−0.016	0.04	3	0.019	3
B. A549 Potencies						
SiO_2_	0.16	−0.003	0.08	1	0.037	1
EHC	0.12	−0.020	0.07	2	0.036	2
TiO_2_	0.09	−0.02	0.05	3	0.029	3

a Magnitude of individual potency estimates derived from the dose–response curves for the two cytotoxicity assays were averaged to yield the overall cytotoxicity‐based potency estimate.

b Magnitude of individual potency estimates derived from the dose–response curves for statistically significant peptides were averaged to yield the overall proteomic potency estimate.

### 3.3 Optimization of cellular proteomic analysis

#### 3.3.1 Protein collection and detergent removal

**Scheme 1:**
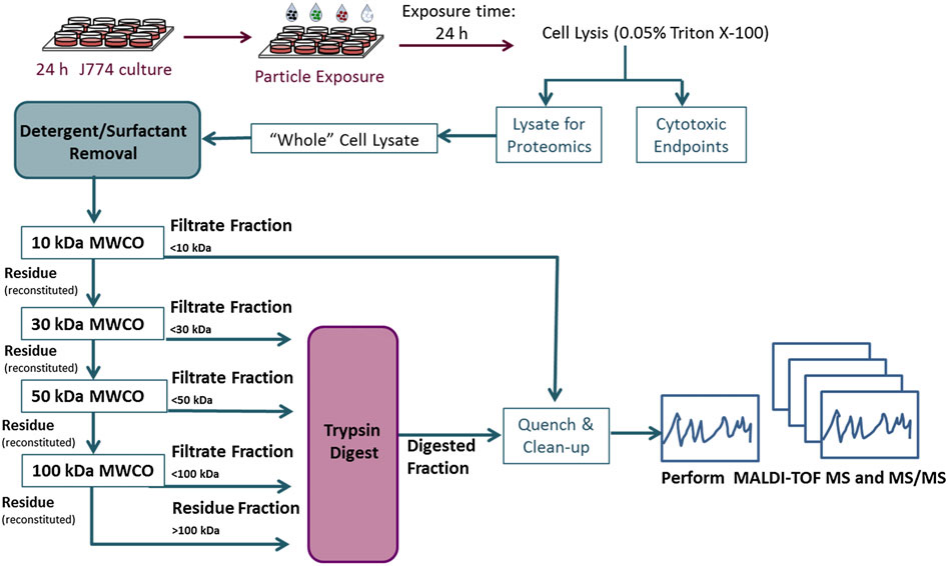
In vitro exposure of cells (J774 and A549) to particles and integrated toxico‐proteomic analyses. MALDI‐TOF MS, matrix‐assisted laser desorption ionization/time of flight/mass spectrometry; MS/MS, tandem mass spectrometry; MWCO, molecular weight cut‐off filter [Colour figure can be viewed at http://wileyonlinelibrary.com]

The final optimized sample preparation scheme is shown in Scheme 1, highlighting the MWCO fraction analyzed in this work and the enzyme combination used for the proteomic analysis. To determine optimal peptide content, cell lysis was carried out either using 0.05% Triton X‐100 or freeze/thaw lysis in dH_2_O. Cell lysis using Triton X‐100 detergent yielded increased protein content compared to the other process, providing relatively more MS information (Supporting information, Figure S1). Direct fractionation of the cell lysate using only a series of MWCO filters was not compatible with MALDI‐TOF‐TOF‐MS due to the presence of Triton X‐100 (0.05% in the lysis buffer) in these samples (Supporting information, Figure S2A, top panel). Of the two different approaches tested to minimize the influence of Triton X‐100 in the cell lysates on MS signals, for the dilution method (A) four sequential dilutions were required to reduce the detergent concentration to a limit above which it would interfere with the MALDI analysis (Method A; Supporting information, Figure S2A, bottom panel). While this method was simple and economical, it did increase the processing time (by ~1.5 hours), which was not ideal. Method B that employed a detergent‐removal spin column was thus selected for all subsequent analysis. These spin columns effectively removed the Triton X‐100 (Supporting information, Figure S2B, top panels), without affecting the peptide signals (Supporting information, Figure S2B, bottom panels).

#### 3.3.2 Protein fractionation

**Figure 3:**
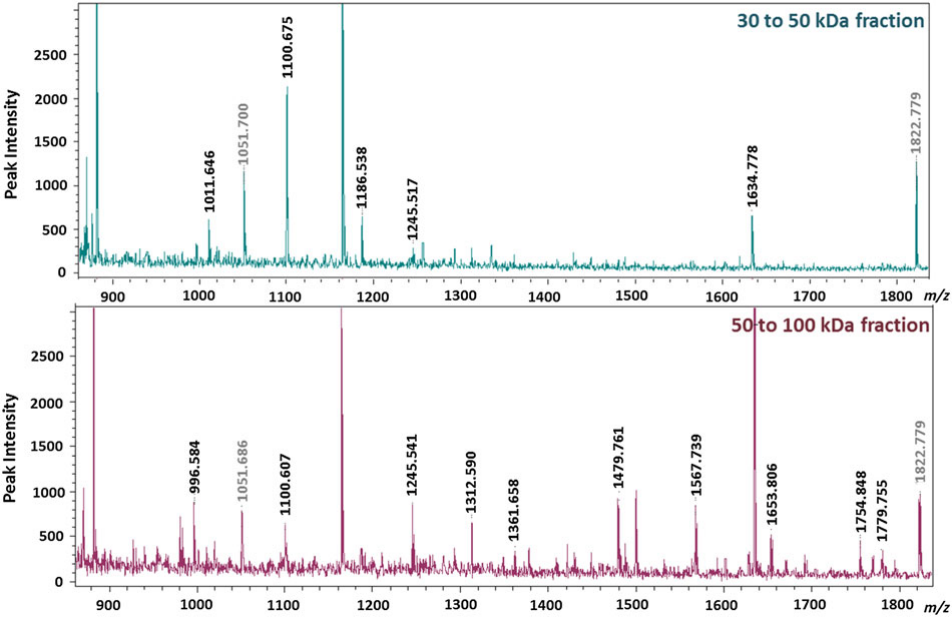
Tryptic peptide analysis results for the different molecular weight cut‐off fractions of cell lysates. Reference peaks associated with the trypsin enzyme have been greyed [Colour figure can be viewed at http://wileyonlinelibrary.com]

Optimization of the MWCO filtration strategies was achieved by assessing three different MWCO fractionation strategies: five, three or four filters. Based on the mass spectra, the four filter approach resulted in the greatest combination of peptide resolution and processing speed. Within this approach, the 50–100 kDa fraction (Figure 3, bottom panel) resulted in the greatest number of tryptic peptide peaks with high signal/noise ratios in the mass spectral scan compared to the 10–30, 30–50 and the >100 kDa fractions. The 30–50 kDa fraction offered the second largest number of peaks with high signal‐to‐noise ratios (Figure 3, top panel). Of the three MWCO filter wash methods assessed to reduce the MWCO filtrate volume and thus subsequent evaporation time, direct wash with only 10% ACN in dH_2_O (total filtrate volume: 200 μl) resulted in minimal change in MS data quality, while decreasing sample processing time by at least 1 hour, when compared to a two‐step wash using dH_2_O followed by 10% ACN/dH_2_O (total filtrate volume: 275 μl). Meanwhile, the one‐step wash with 50 mm ammonium acetate lowered analyte yields and mass spectral intensities (data not shown).

#### 3.3.3 Enzyme digestion

**Figure 4:**
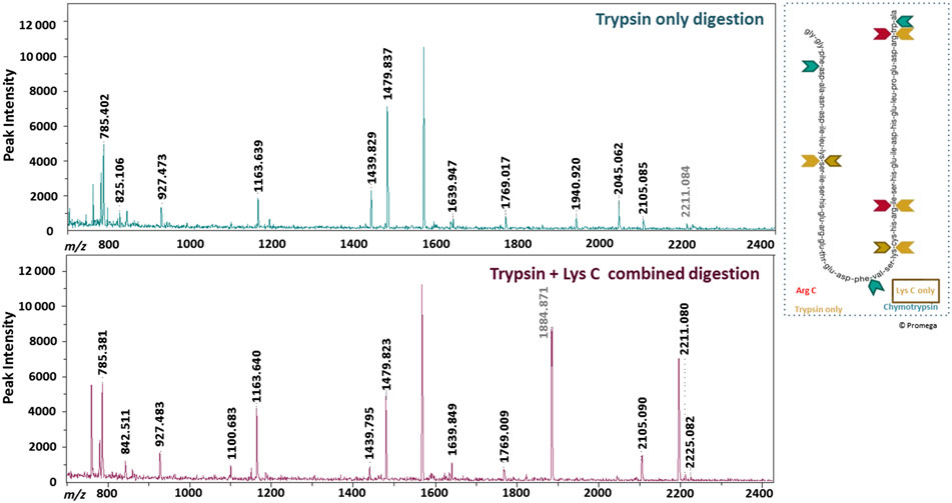
Effect of digestion enzyme type on mass spectral information. Reference peaks associated with the trypsin enzyme have been greyed [Colour figure can be viewed at http://wileyonlinelibrary.com]

Results from the optimization of enzyme digestion efficiency showed that increased number of peptides and peak intensities were observed in the mass spectral scan by conducting an overnight incubation with trypsin combined with Lys C as opposed to trypsin alone (Figure 4), while chymotrypsin resulted in a lower number of cuts than trypsin alone (data not shown). Of the enzyme digestion buffers tested, the more alkaline 50 mm Tris–HCl, pH 8.0 buffer performed poorly compared to the more neutral 50 mm NH_4_OAc, pH 7.4, which generated greater peptide numbers with increased peak intensities (Supporting information, Figure S3).

#### 3.3.4 MALDI‐TOF‐TOF‐MS analyses

Greater number of peptides with increased peak intensities were achievable on manual execution of the MALDI‐TOF‐TOF MS analysis compared to the automated run (Operating condition 1 shows the optimized automated run Supporting information, Figure S4, top panel; Operating condition 2 shows the optimized manual analyses, Supporting information, Figure S4, bottom panel). Optimized settings for analysis were determined to be a gain of 9.5, pulsed ion extraction of 80 seconds, and 2000 laser shots. The analytical repeatability of this process was also high following this optimization. Relative standard deviation was less than 20% for the majority (>89%) of the peptides detected, including for biological replicates. These analytical precision values were consistent across a broad *m*/*z* range (700–3100 *m*/*z*).

### 3.4 Real sample analysis using the optimized method

**Figure 5:**
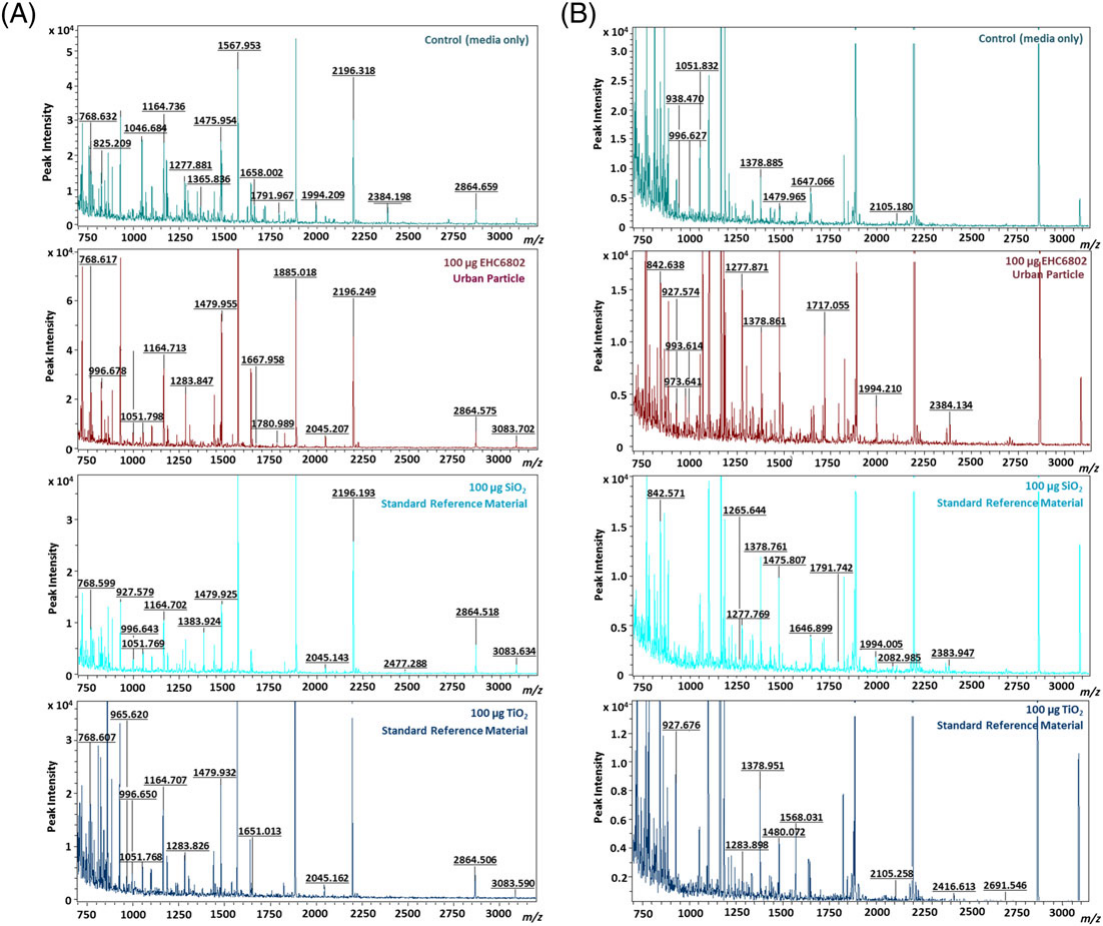
Proteomic profiles of lysates of cells after particulate exposure: (A) J774 cells; (B) A549 cells [Colour figure can be viewed at http://wileyonlinelibrary.com]

**Figure 6:**
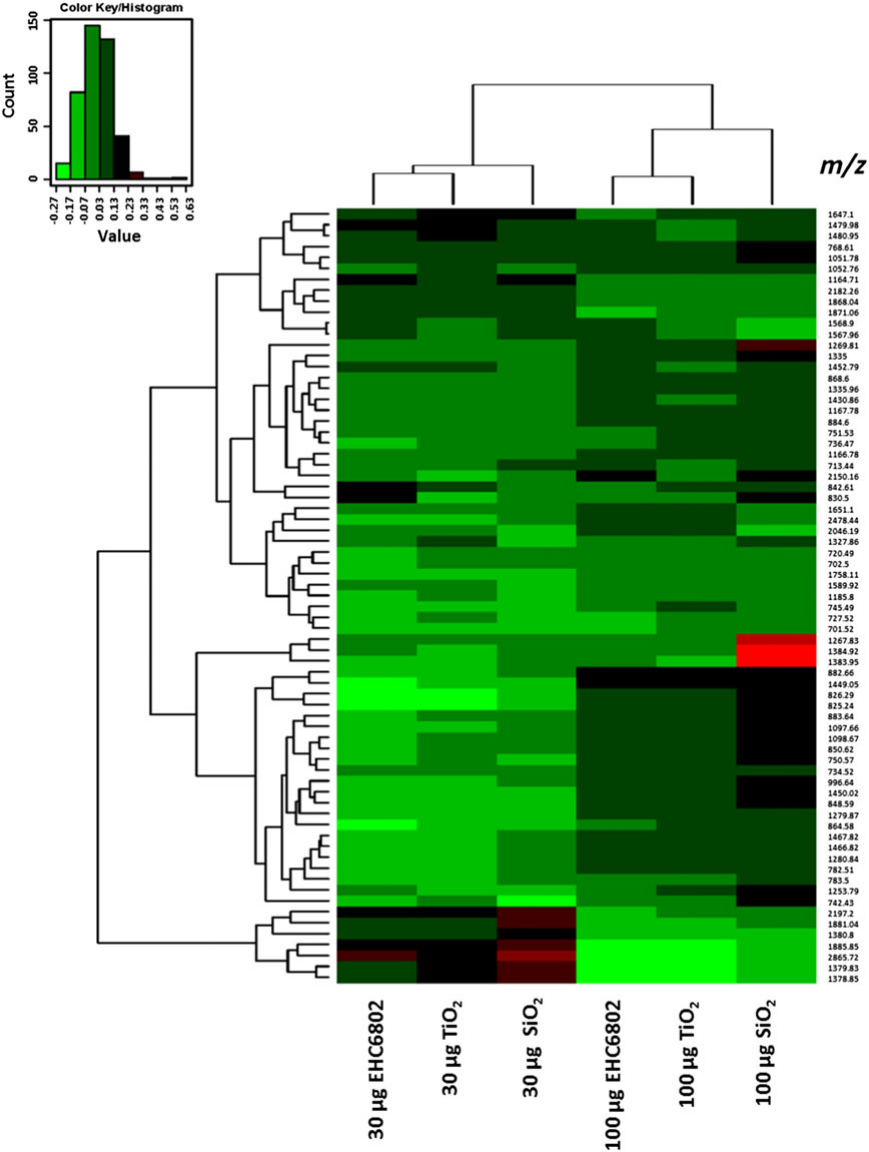
Heat map and hierarchical clustering of particle‐specific changes in J774 tryptic peptides. Green, downregulation; red, upregulation

**Figure 7:**
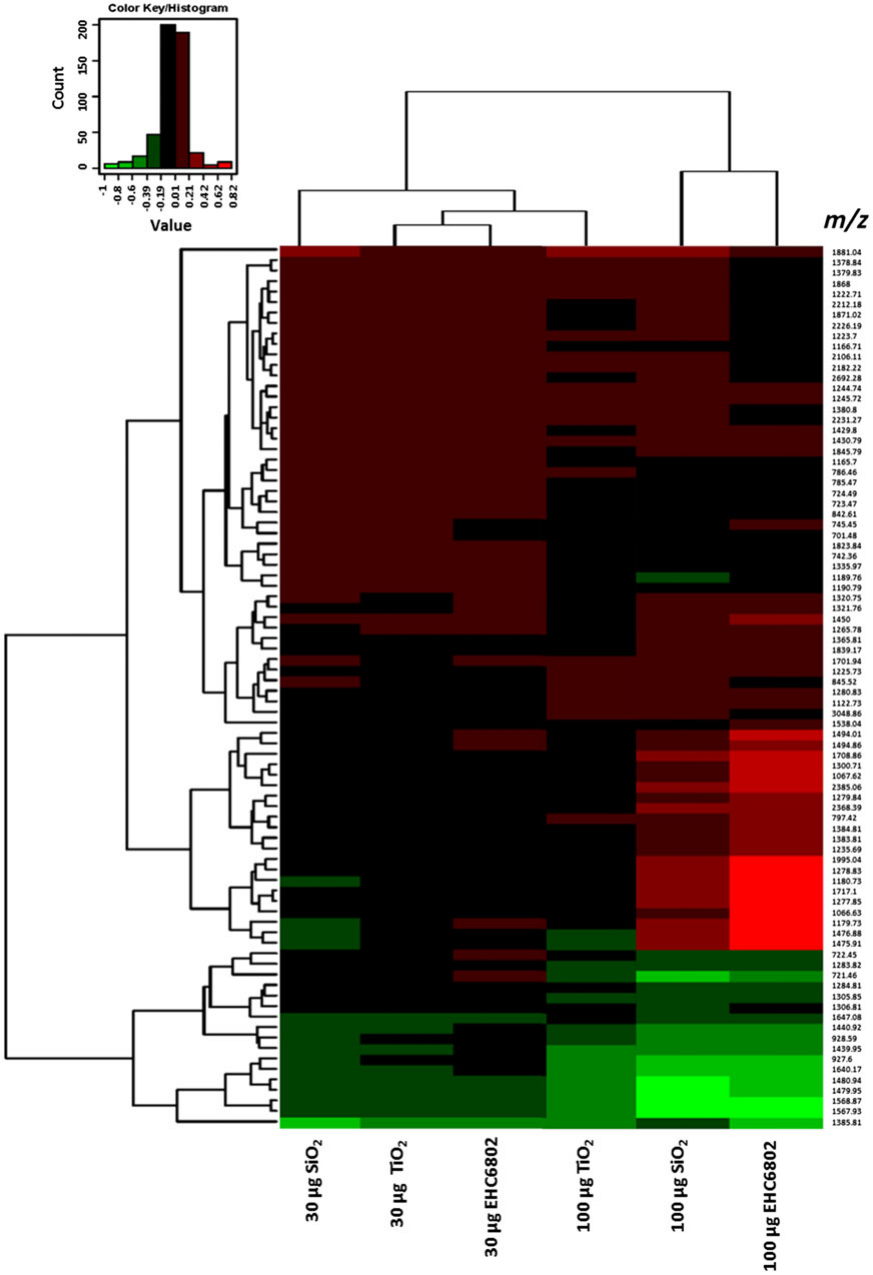
Heat map and hierarchical clustering of particle‐specific changes in A549 tryptic peptides. Green, downregulation; red, upregulation

Representative mass spectral scans following exposure to 100 μg ml^−1^ dose of each particle are shown for J774 (Figure 5A) and A549 cells (Figure 5B). Comparison of the peptide peaks revealed particle‐ and dose‐dependent changes in the proteome of both cell types. Hierarchical clustering of peptides identified as significant by ClinPro Tools software analyses confirmed the cytotoxicity data. Protein expression profile for SiO_2_ (highest toxicity based on cytotoxicity assay results) was distinct from the protein expression profiles for the other two particle exposures (EHC6802 and TiO_2_; Figure 6, J774 and Figure 7, A549). Furthermore, for J774 cells, clustering of tryptic‐peptide patterns revealed dose‐dependent differences in protein changes with the different PM exposures. Upon performing paired *t*‐tests on the top 200 peptides based on signal to noise ratio (S/*N* > 3), 106 statistically different peaks were detected for J774 and 88 for A549 cells. The comparison between the relative potency rankings based on classical cytotoxicity results and based on selected statistically significant protein changes are listed in Table 3. The β (potency estimate) value for J774 cells exposed to SiO_2_ particles was the highest for both cytotoxicity and proteomics‐based analyses. Similarly, with A549 cells, cytotoxicity and proteomic endpoints‐based potency estimates were greatest with SiO_2_ exposures and lowest for cells exposed to TiO_2_.

## 4 DISCUSSION

The toxicity of PM can be attributed to its physicochemical properties that are influenced by source emissions and atmospheric conditions (Kelly & Fussell, 2012; Rohr & Wyzga, 2012; Stanek, Sacks, Dutton, & Dubois, 2011). Identification of PM exposure‐induced in vitro cellular toxicity mechanisms and their association with PM characteristics can be useful in risk analysis (Grahame & Schlesinger, 2007).

As indicated by SEM, EHC6802, SiO_2_ and TiO_2_ comprise of particles of relatively large size (μm dimensions). The elemental analysis revealed that the particles contained metals that can be bioavailable. In addition, EHC6802 is an urban air particle and thus contains various chemical species including metals, whereas, SiO_2_ and TiO_2_ are reference particles and are seen to contain some other trace level metals as well. These components may potentially contribute to cytotoxicity. All three particles exhibited low stability in liquid media, with increased tendency to agglomerate/aggregate and sediment, as substantiated by DLS and zeta potential analysis. The data suggest that wide‐scale deposition of particles on cell surface is expected during the relatively short period of cell exposure. It is notable that the DLS analysis results should be interpreted carefully because of the large particle sizes and the heterogeneity of the size distributions for all three particles. The DLS analysis also revealed the presence of particles or components (200–500 nm in size), which remained dispersed in cell exposure medium, representing a fraction of particles that could be internalized by cells. As shown here, PM dynamics in cell culture media are likely an additional determinant of the cellular response to particles.

The environmental particle collection on filters can result in small amounts of particle mass, and this can influence the downstream analytical processes. In this work, the use of small particle mass for in vitro exposure experiments necessitated the establishment of an integrated cytotoxicity/toxicoproteomic platform that can provide samples for both classical cytotoxicity and proteomic endpoint analyses from a 96‐well plate exposure experiment (Scheme 1).

Our cellular cytotoxicity assay results exhibited increased LDH release (Figure 2C,D) after high‐dose PM exposures, for both cell types. LDH release into the cell culture supernatants suggested that particle exposures affected cell membrane permeability and thus potential subsequent cell death (Chan, Moriwaki, & De Rosa, 2013). For J774 cells, both resazurin reduction and LDH release assays consistently revealed that the exposure of cells to silica particles (SiO_2_) and EHC6802 (urban air PM) relatively increased cell‐PM reactivity compared to TiO_2_ (Figure 2). For A549 cells, the cytotoxicity assay results at the cellular level appeared to be assay type‐dependent (Figure 2). It is well known that the nature of cell–PM interactions can differ for epithelial vs. macrophage cells (Kumarathasan et al., 2015). The extent of cytotoxic response to particles likely results from a combination of cell surface‐induced damage by particles (as indicated by the LDH release data) and cytotoxicity induced through uptake of particles or their components by the macrophages and epithelial cells.

For proteomic processing, only the 0, 30 and 100 μg cm^−2^ samples were selected due to the frank cytotoxicity observed at the 300 μg cm^−2^ dose based on cytotoxicity results, and the high likelihood for this dose to be associated with mostly cell death mechanisms (Vuong et al., 2017). The goal of this study was not to identify, exhaustively and comprehensively, the entire cellular proteome following exposure to airborne particles, but rather to develop a proteomic method that will generate information with an acceptable sensitivity and specificity to conduct toxicity screening for environmental PM in small volumes of in vitro cell lysate samples. We previously demonstrated the application of shot‐gun MS‐based proteomic analysis using MALDI‐TOF‐TOF‐MS methods (Kumarathasan et al., 2014; Mohottalage, Vincent, & Kumarathasan, 2009) to discriminate biologically different samples. The current work was thus performed on this analytical platform to assess complex in vitro PM toxicity characteristics, after optimization of various sample preparation and MS analysis stages to obtain the best subset of high‐content, meaningful proteomic information. While we were able to isolate approximately 700 tryptic peptide peaks with S/*N* > 2 from the peptide mass fingerprint for each cell type, for the purposes of this work, the top 200 peaks (based on S/N ratio and spectral intensities) were subjected to further analysis.

The optimization of cell lysate sample preparation procedures was aimed at (1) shortening sample processing time, and (2) to enhance the number of peptide peaks that are resolved and are of high intensities. The MS analyses results of the different molecular weight fractions identified the 50–100 kDa fraction to exhibit greatest number of resolved peaks with increased analyte intensities. These MS profiles had 58% more peptide peaks than the 30–50 kDa (Figure 3) fraction, which was the fraction that yielded second greatest high content analyte information, of all fractions tested. We therefore chose the 50–100 kDa fraction in this work to screen for cellular proteomic changes due to in vitro PM exposures.

The combination of trypsin enzyme with Lys‐C for the digestion of cellular proteins was effective in increasing the number and intensity of peptides observed. This improved digestion efficiency was attributed to more complete cleavage at lysine residues of the protein by the addition of Lys‐C, as trypsin alone misses approximately 20% of all lysine cleavage sites (Saveliev et al., 2013). In addition, although the manufacturer recommended the use of 50 mm Tris–HCl (pH 8.0) as the digestion buffer, greater peptide numbers and increased peptide intensities were obtained with 50 mm ammonium acetate (pH 7.4) as the digestion buffer (Supporting information, Figure S3), an observation that is in line with previous results (Kumarathasan et al., 2005). In addition, we used ProteaseMax to enhance the enzymatic digestion that was followed by quenching with TFA and clarification by centrifugation to optimize further the process by removal of the degradation products of ProteaseMax, if formed.

The comparison of MS profiles of tryptic digests of cell lysates obtained through lysing of cells using freeze–thaw cycles with dH_2_O, or using Triton X‐100 detergent showed that the latter approach gave relatively more MS information (Supporting information, Figure S1). The increase in tryptic peptide signals and intensities with Triton X‐100 lysis perhaps can be attributed to increased solubilization of membrane proteins and decreased hydrophobic protein losses during the initial sample processing. This supported the use of Triton X‐100 in the in vitro toxicoproteomic procedure. The consequence of an integrated toxicoproteomics and cellular cytotoxicity endpoint analysis, which utilized simultaneous collection of samples for cytotoxicity and proteomics from the same cell culture plate was the small protein content available for proteomic analysis (e.g., 20 μg μl^−1^). Yet, the optimization of sample preparation led to ~15% increase in the number of observed analyte peaks in the MS scan and ~2‐fold increase in peak intensities as opposed to a non‐optimized method.

In an effort to decrease sample processing time during the pre‐fractionation of cell lysates, MWCO filtration process was optimized. Our findings suggested that the four‐filter fractionation strategy was the best compromise between reducing the complexity of protein mixture and speed of sample processing. In addition, the use of one‐step MWCO filtrate collection using 10% ACN (aq) minimized the number of centrifugation stages and the subsequent drying time without compromising observable peptide signals during the MS analyses. All stages of the sample preparation procedure described here are amenable to automation using fluid handling robotics to enable further enhancement of analytical reproducibility with unattended sample processing making the process suitable for high‐throughput analyses. In terms of instrumental operation conditions, we noted that operator intervention assisted in observing relatively larger number of peptides (*m*/*z* values) compared to the automated option (Supporting information, Figure S4). This may be partly attributed to the fixed nature of sampling (e.g., selected random walk pattern) of the MALDI spot as well as the selection of parameters in this mode.

**Figure 8:**
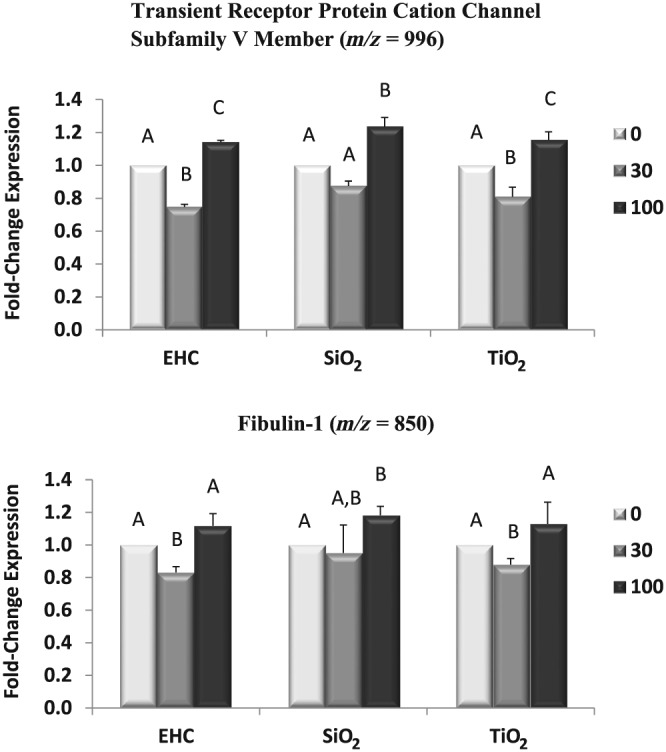
Profiles of J774 cell proteins after exposure to particles exhibiting biphasic responses with significantly (*P* < 0.05) different dose‐related changes. (a) Transient receptor potential cation channel subfamily V member. (b) Fibulin‐1. Note: significantly different doses are labelled differently, e.g. A is significantly different from B

Application of the optimized toxicoproteomics method in the assessment of in vitro PM effects led to the observation of statistically significant (*P* < 0.05) protein changes even with PM exposures at low doses (30 μg cm^−2^) compared to the control (Figure 8). Meanwhile, in terms of the classical cytotoxicity assays, PM toxicity‐related changes reached significance (*P* < 0.05) only at high‐dose exposures (Figure 2).

Heat map and hierarchical clustering analysis results on tryptic peptide profiles (*P* < 0.05) of PM exposed vs. control cell lysates based on ClinPro Tools software analysis output showed PM exposure‐related protein changes. Using this approach, we have demonstrated particle‐induced molecular level changes, for both cell lines. For J774, clustering is seen with respect to PM exposure dose (30 vs. 100 μg), and based on PM type, such as the clustering of EHC6802 and TiO_2_ as opposed to SiO_2_ (Figure 6). For A549 cells, EHC6802 and TiO_2_ clustered together at the low dose, but at the high dose EHC6802 clustered with SiO_2_ (Figure 7). This suggests cell type‐specific differences in protein profiles, perhaps due to differences in PM–cell interactions.

Potency estimates (β) for the different particles employed in this study were calculated based on fold‐effects of PM exposure‐related cytotoxicity or proteomic responses compared to their control levels, for each cell line, using a previously reported method (Vincent, Goegan, et al., 1997). Interestingly, PM toxicity ranking for each cell type, based on average potency estimates derived from both classical cytotoxicity assay values, was as follows: SiO_2_ > EHC6802 > TiO_2_, irrespective of the cell type (Table 3). The order of the PM toxicity ranking remained the same when the average potency estimates were calculated using the significantly (*P* < 0.05) altered tryptic peptides, for both cell types, demonstrating the consistency between these two approaches (Table 3).

Although, the primary aim of this work was to develop high‐content MS‐based proteomic profiling for PM‐related changes and for reliable assessment of relative PM potencies, nevertheless, we tentatively identified some (false discovery rate < 5% with at least one unique tryptic peptide) candidate biomarkers of PM exposures for both cell types. The candidate marker proteins identified in these cell types were associated with cell proliferation, apoptosis, energy metabolism, membrane and mitochondrial protein changes in line with what we have observed before (Vuong et al., 2017). For instance, one of the identified candidate markers of PM exposure in J774 cells was caspase‐7, which is associated with a mitochondrial apoptosis pathway (Bastiani, Vidotto, & Horn, 2005). The other mitochondrial apoptosis‐related marker identified in these cells was cytoplasmic polyadenylation element‐binding protein‐1. A previous work by Dagher et al. (2006) has shown that PM2.5 exposure induced apoptosis by activating the mitochondrial pathway. Fibulin‐1 is another candidate marker identified in J774 cells and is known to play a role in cell adhesion, and is reported to be present in atherosclerotic plaques, which contain macrophages and may be of relevance, as PM exposure has been shown to be associated with atherosclerosis progression (Argraves et al., 2009). Some of the mitochondrial energy metabolism‐related proteins identified in J774 include DnaJ homolog subfamily B member 1 and mitochondrial ATP synthase subunit O. In addition, H‐2 class 1 histocompatibility antigen D‐P alpha chain protein was identified in J774 cells, which is known to be associated with activation of macrophages (Zur Lage, Goethe, Darji, Valentin‐Weigand, & Weiss, 2003). Similarly, PM exposure‐related marker proteins identified in A549 cells included fibroblast growth factor receptor 3, zinc finger proteins, monocarboxylate transporter 4, E3 ubiquitin protein ligase RNF31 and NACHT, LRR and PYD domains containing protein 1. These proteins are involved in cell proliferation, apoptosis, cell membrane transport, energy metabolism and inflammation‐related responses. (Granja et al., 2015; Kong et al., 2015; Li et al., 2016; Qi et al., 2017). Furthermore, these findings reveal that the observed proteomic changes are consistent with cytotoxicity results. Our results demonstrate that although classical cytotoxicity data are useful in rapid screening for PM toxicity by following changes in cell metabolism and death, the high‐content MS‐based proteomic information can offer greater resolution in terms of molecular level changes for the identification of reliable dose–response relationships and toxicity mechanisms.

The physicochemical composition of PM can vary depending on sources, and so can the associated toxicity characteristics. To keep abreast with the risk estimation for such PM exposures, a methodology that can offer faster analysis of multiple control and treatment samples alongside to minimize day‐to‐day variation in the analysis results, will be an ideal choice. The optimized toxicoproteomic method in this work yielded high‐content information on protein changes with relatively small protein contents, requires no solvent, enables fast analysis with potential for automation and complements classical cytotoxicity assays, and thus fits the integrated in vitro PM toxicity screening schedule.

## 5 CONCLUSION

The integrated, in vitro toxicoproteomic pipeline has the capacity to provide high‐content data on PM cytotoxic reactivity‐related molecular level information. Toxicoproteomic data can validate classical cytotoxicity results and provide a complementary mechanistic insight into in vitro PM effects. Thus the integrated in vitro PM toxicity testing approach can be valuable in future particle toxicology studies.

## CONFLICT OF INTEREST

The authors do not have any conflict of interest.

## Supporting information

Figure S1 Supporting information

Figure S2 Supporting information

Figure S3 Supporting information

Figure S4 Supporting information
