# Supplementary figures and images for: A matrix‐assisted laser desorption ionization–time‐of‐flight–time‐of‐flight–mass spectrometry‐based toxicoproteomic screening method to assess in vitro particle potencies
Source: J Appl Toxicol. 2018 May 29;38(10):1302–15. doi: 10.1002/jat.3642 (PMC6220844; doi:10.1002/jat.3642)

- Figure S1:


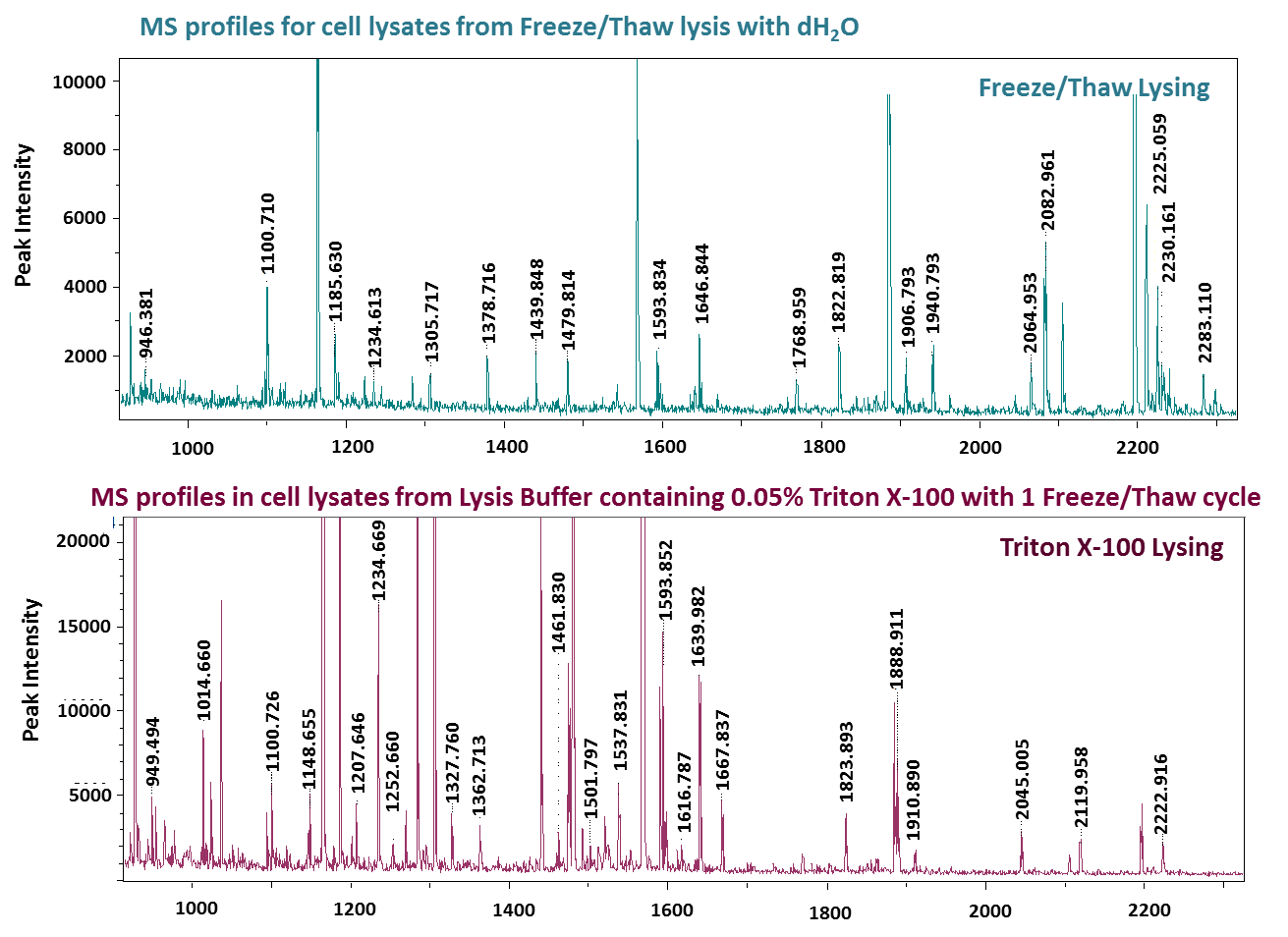

Supplement: Supplementary file 1 — Figure S1 Supporting information [file JAT-38-1302-s001.docx]

- Figure S2:


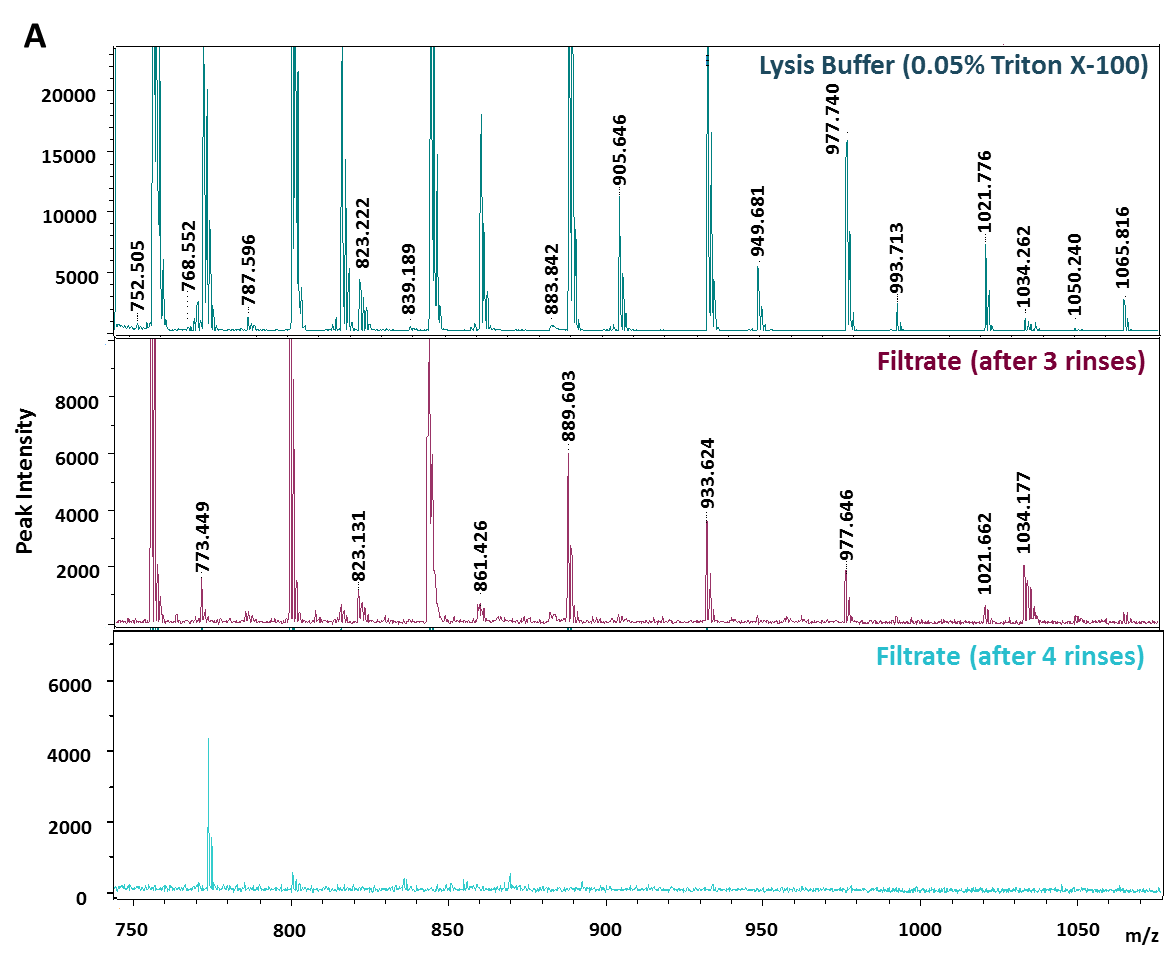


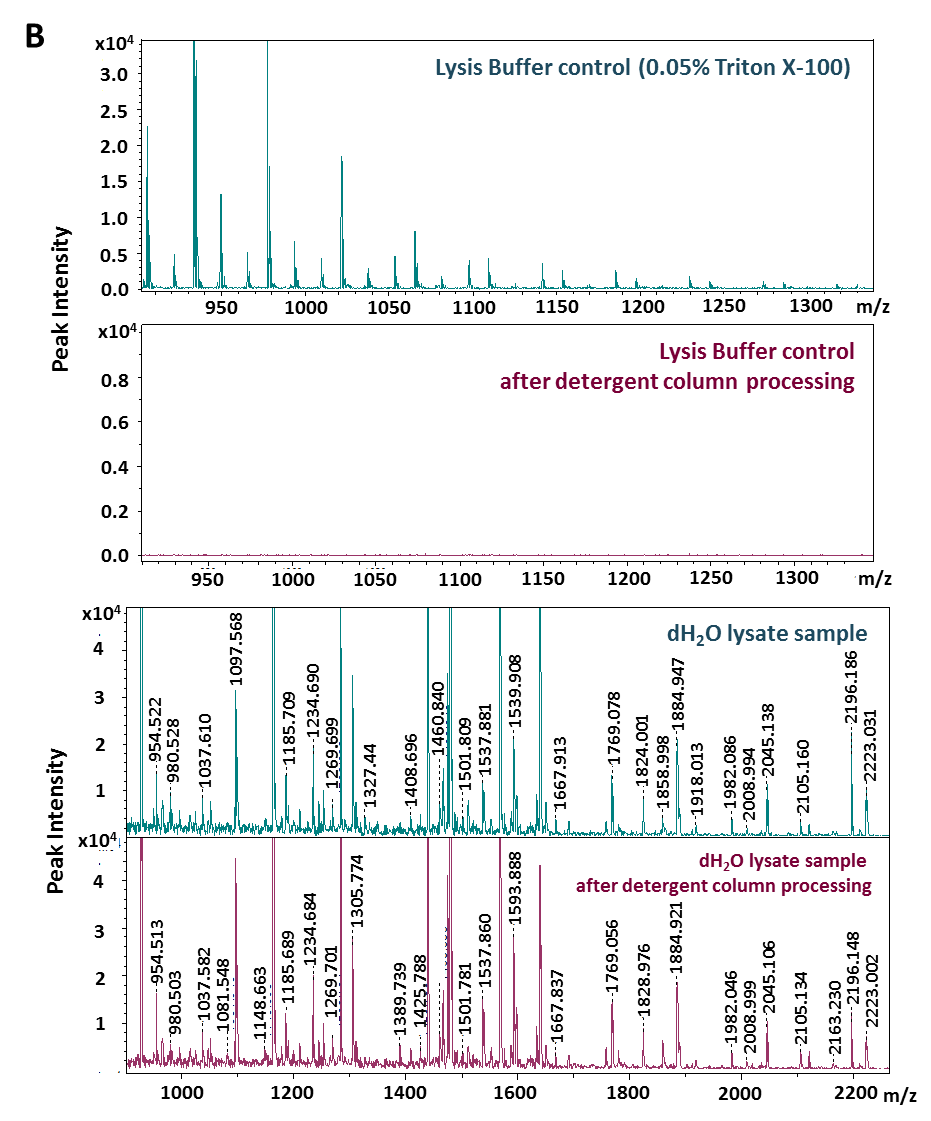

Supplement: Supplementary file 2 — Figure S2 Supporting information [file JAT-38-1302-s002.docx]

- Figure S3
-
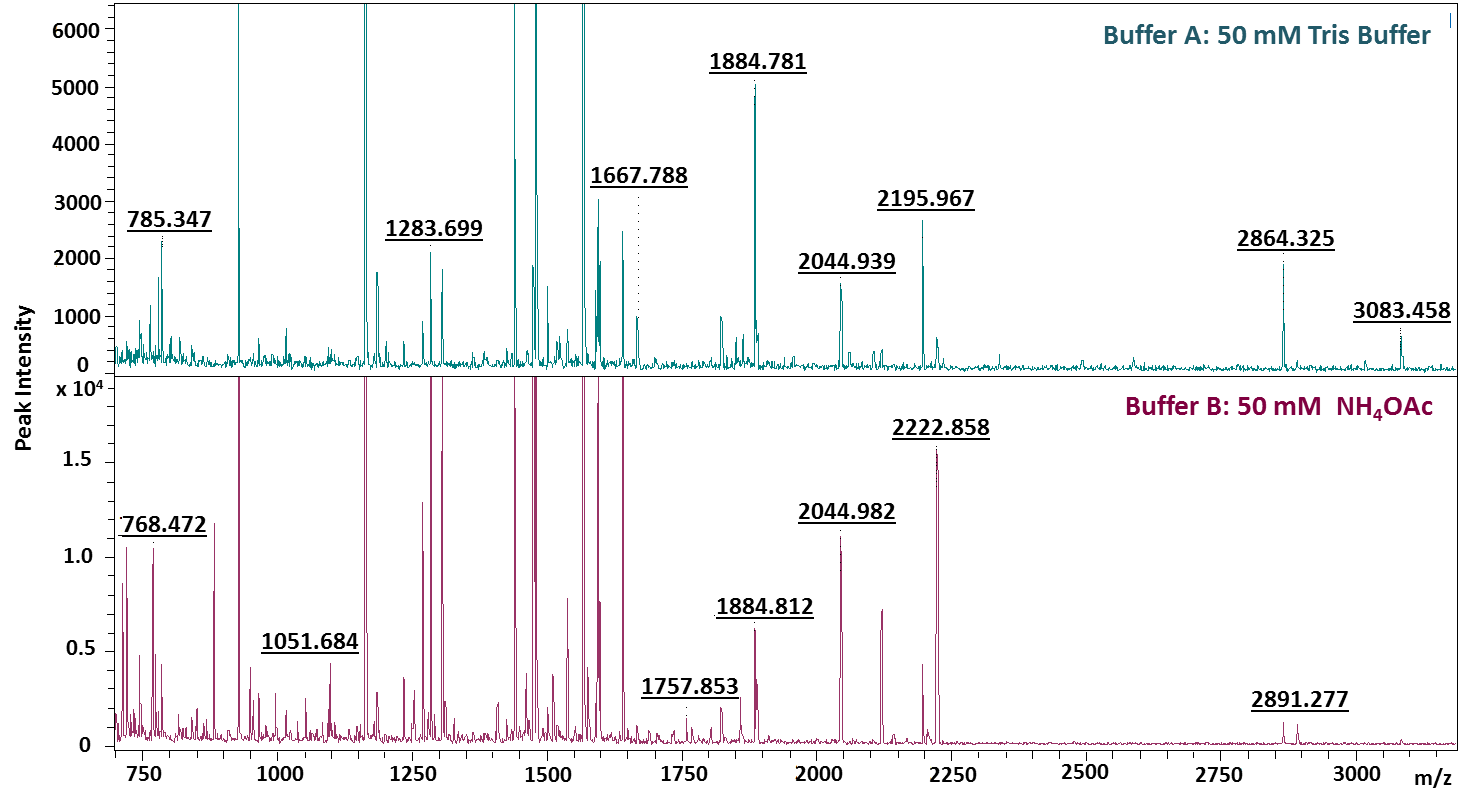

Supplement: Supplementary file 3 — Figure S3 Supporting information [file JAT-38-1302-s003.docx]

- Figure S4


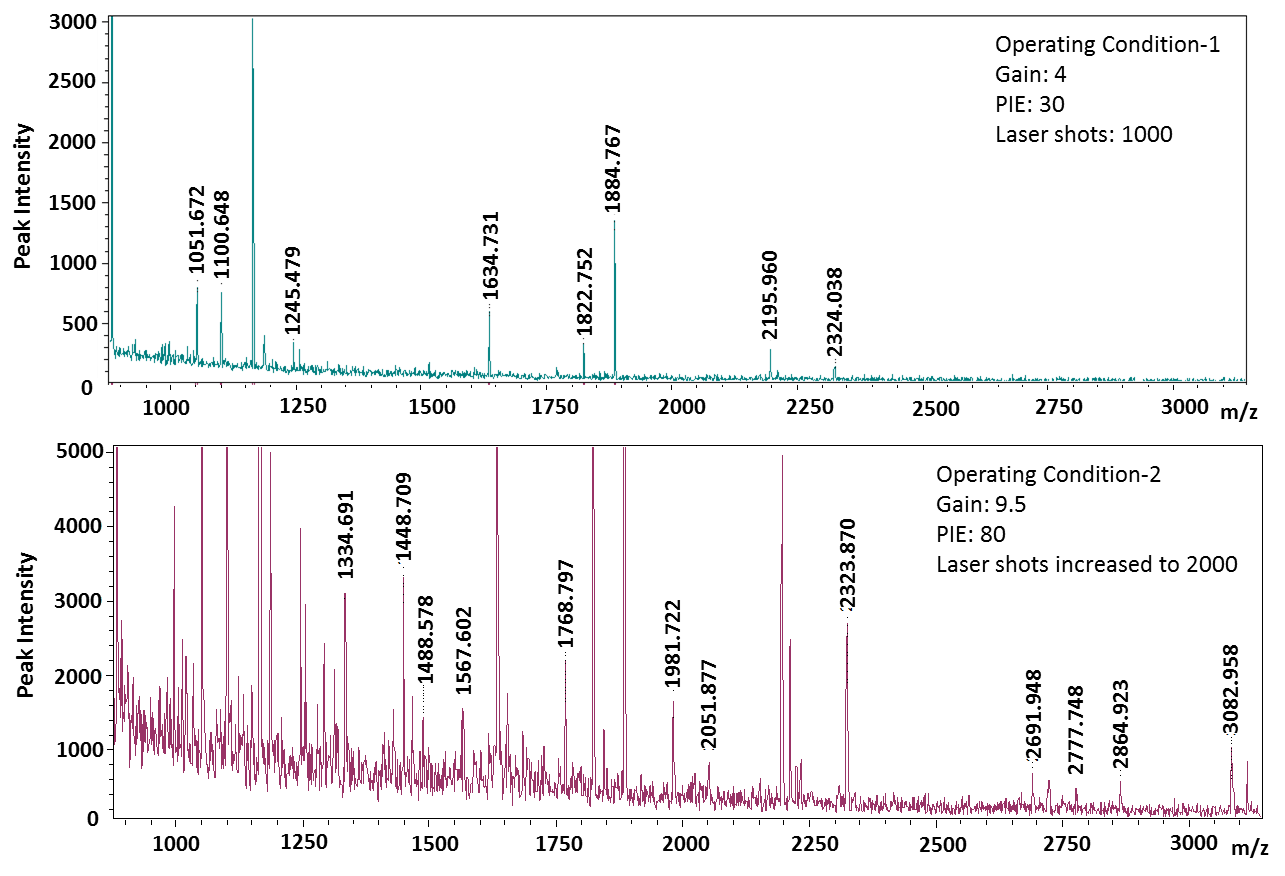

Supplement: Supplementary file 4 — Figure S4 Supporting information [file JAT-38-1302-s004.docx]
